# Systematic comparison of hUC-MSCs at various passages reveals the variations of signatures and therapeutic effect on acute graft-versus-host disease

**DOI:** 10.1186/s13287-019-1478-4

**Published:** 2019-11-28

**Authors:** Qinjun Zhao, Leisheng Zhang, Yimeng Wei, Hao Yu, Linglin Zou, Jiali Huo, Hongju Yang, Baoquan Song, Teng Wei, Dan Wu, Wenxia Zhang, Lei Zhang, Dengke Liu, Zongjin Li, Ying Chi, Zhibo Han, Zhongchao Han

**Affiliations:** 1 State Key Laboratory of Experimental Hematology, Institute of Hematology & Blood Diseases Hospital, Chinese Academy of Medical Sciences & Peking Union Medical College, 288 Nanjing Road, Tianjin, 300020 China; 2 National Stem Cell Engineering Research Center, Tianjin Ang-sai Stem Cell and Gene Technology Co., Ltd., Tianjin, 300450 China; 3 The Postdoctoral Research Station, School of Medicine, Nankai University, Tianjin, 300071 China; 4 The Enterprise Postdoctoral Working Station, Tianjin Chase Sun Pharmaceutical Co., Ltd., Tianjin, 301700 China; 5 Precision Medicine Division, Health-Biotech (Tianjin) Stem Cell Research Institute Co., Ltd., Tianjin, 301700 China; 6 Jiangxi Research Center of Stem Cell Engineering, Jiangxi Health-Biotech Stem Cell Technology Co., Ltd., Shangrao, 334000 China; 7 Division of Gastroenterology, The First Affiliated Hospital of Kunming Medical University, Kunming, 650032 China; 8 Department of Oncology, Affiliated Hospital of Southwest Medical University, Luzhou, 646000 China; 9 Jiangsu Institute of Hematology, The First Affiliated Hospital of Soochow University, Suzhou, 215006 China; 10 Cytotherapy Laboratory, Shenzhen People’s Hospital & The second Clinical Medical College of Jinan University, Shenzhen, 518020 China

**Keywords:** UC-MSCs, Various passages, Hematopoiesis, Graft-versus-host disease

## Abstract

**Background:**

Mesenchymal stem cells are heterogenous populations with hematopoietic supporting and immunomodulating capacities. Enormous studies have focused on their preclinical or clinical therapeutic effects, yet the systematic study of continuous in vitro passages on signatures and functions of UC-MSCs at both the cellular and molecular levels is still lacking.

**Methods:**

In this study, to systematically evaluate the biological properties of MSCs at various passages, we analyzed biomarker expression, cell proliferation and apoptosis, chromosome karyotype, and tri-lineage differentiation potential. Subsequently, we took advantage of whole-exome sequencing to compare the somatic hypermutation of hUC-MSCs at P3, P6, and P15 including SNV and INDEL mutations. In addition, to explore the safety of the abovementioned hUC-MSCs, we performed metabolic pathway enrichment analysis and in vivo transplantation analysis. Furthermore, we cocultured the abovementioned hUC-MSCs with UCB-CD34^+^ HSCs to evaluate their hematopoietic supporting capacity in vitro. Finally, we transplanted the cells into acute graft-versus-host disease (aGVHD) mice to further evaluate their therapeutic effect in vivo.

**Results:**

The hUC-MSCs at P3, P6, and P15 showed similar morphology, biomarker expression, and cytokine secretion. hUC-MSCs at P15 had advantages on adipogenic differentiation and some cytokine secretion such as IL-6 and VEGF, with disadvantages on cell proliferation, apoptosis, and osteogenic and chondrogenic differentiation potential. Based on the SNP data of 334,378 exons and bioinformatic analyses, we found the somatic point mutations could be divided into 96 subsets and formed 30 kinds of signatures but did not show correlation with risk of tumorigenesis, which was confirmed by the in vivo transplantation experiments. However, hUC-MSCs at P15 showed impaired hematologic supporting effect in vitro and declined therapeutic effect on aGVHD in vivo.

**Conclusions:**

In this study, we systematically evaluated the biological and genetic properties of hUC-MSCs at various passages. Our findings have provided new references for safety and effectiveness assessments, which will provide overwhelming evidence for the safety of hUC-MSCs after continuous in vitro passages both at the cellular and molecular levels for the first time. Taken together, our studies could help understand the controversial effects of disease treatment and benefit the clinical research of UC-MSCs.

## Background

Mesenchymal stem cells are acknowledged as the most important niche cells and have broad prospects for regenerative medicine, which mainly due to their unique attributes such as self-renewal, multi-lineage differentiation potential, and hematopoietic supporting effect and immunomodulating function [1–4]. The cell population was firstly isolated from the bone marrow in the 1960s and followed by other stromal fractions of adult tissues such as the adipose tissue, umbilical cord, placenta, dental pulp, synovial membrane and perivascular, and even human pluripotent stem cells (hPSCs) [5–8]. Thus, due to the wide range of sources for MSC extraction and preparation and the lack of unique biomarker, for decades, MSCs are recognized as a cell population with significant heterogeneity [4, 9]. Until the year of 2006, the International Society for Cellular Therapy (ISCT) released a professional standard for MSCs with the golden criterions: spindle shape and adherent growth; CD73, CD90, and CD105 expression; and adipogenic, osteogenic, and chondrogenic differentiation [4, 10].

To date, according to the ClinicalTrials.gov website of NIH, a total number of 983 clinical studies have been registered for series of disease treatment, such as hematological malignancies, acute-on-chronic liver failure, acute severe respiratory failure, type I and II diabetes, psoriasis, graft-versus-host disease (GVHD), cerebral palsy, ulcerative colitis, and even wound healing [11–15]. Meanwhile, several preclinical studies on disease treatment are in process as well, including acute myocardial infarction (AMI), rheumatoid arthritis (RA), osteoarthritis, critical limb ischemia (CLI), and aplastic anemia [5, 16–19]. Of these studies, we and other investigators have indicated the efficacy of human umbilical cord mesenchymal stem cells (hUC-MSCs), which are promising sources without limitation in supply [15, 20, 21]. Moreover, the long-term effectiveness and quality of MSCs on the prognosis of the patients or animals mostly depend on cell vitality and homing ability of the infused cells [22]. Recently, Zhang et al. showed that hUC-MSCs at various passages would result in different therapeutic effects on acute liver failure in rat, which indicated long term in vitro passages could attenuate the biological properties and act as a lingering problem pertaining to MSC-based clinical application [22]. However, the systematic evaluation of in vitro passages on biological characteristics and functions of hUC-MSCs is still lacking, which is urgent for standardizing and guiding therapeutic purposes of MSCs [4, 9].

In this study, to illuminate the influence of serial passages on phenotypic characterization, biological function, and molecular genetics of MSCs, we systematically analyzed hUC-MSCs at passages 3, 6, and 15 (short for P3, P6, P15, respectively). Generally, hUC-MSCs at the abovementioned passages showed similarities in cellular morphology, biomarker expression, chromosome karyotype, and most of the cytokine secretion, together with comparable cumulative mutation spectrum. However, when compared to those in the other groups, hUC-MSCs at the higher passage (P15) exhibited impaired cell proliferation capacity, osteogenic and chondrogenic differentiation potential, and hematopoietic supporting effect, but with enhanced apoptosis and adipogenic differentiation potential in vitro. Furthermore, by conducting a GVHD mouse model, we found hUC-MSCs at P15 showed an attenuated therapeutic effect on acute graft-versus-host disease in vivo.

## Methods

### hUC-MSC culture and passage

The three independent hUC-MSC lines at various passages (P2, P5, and P14) were preserved by our lab using the cryoprotectant (5 × 10^6^ cells in 10% DMSO in MSC culture medium) as we previously reported [23]. The hUC-MSCs (P2, P5, and P14) were simultaneously thawed and cultured in the MSC culture medium (DMEM/F12 basal medium supplemented with 10% fetal bovine serum (Australia), 1% NEAA (Gibco), 1% l-glutamine (Gibco), 4 ng/ml bFGF (PeproTECH), 4 ng/ml EGF (PeproTECH), 1% penicillin and streptomycin (ThermoFisher)). The medium was changed every 3 days. When the abovementioned hUC-MSCs reach 80% confluency, the cells were dissociated with 0.25% Trypsin-EDTA at 37 °C for 5 min and resuspended by hUC-MSC culture medium. Then, the hUC-MSCs were collected by centrifugation at 300×*g* for 5 min. After discarding the supernatant, the cells were resuspended and seeded in the hUC-MSC medium at 37 °C, 5% CO_2_. Finally, the hUC-MSCs at P3, P6, and P15 were prepared. Three days later, the hUC-MSCs were used for the corresponding tests and analyses.

### Flow cytometry analysis

hUC-MSCs at various passages (P3, P6, P15) were dissociated into single cells by 0.25% Trypsin-EDTA (Gibco) and stained with the indicated antibodies against CD3, CD4, CD11b, CD14, CD19, CD25, CD29, CD34, CD44, CD45, CD66b, CD73, CD90, CD105, CD127, HLA-DR, Annexin-V, and 7AAD, in 0.2% BSA for 20 min in the dark. After washing with 1× PBS twice, the cells were analyzed by FACS Canto II (BD Biosciences) as we reported previously [6, 24]. The data were analyzed with FlowJo 7.0 (Ashland). The antibodies were listed in Additional file 7: Table S3.

### Quantitative real-time PCR

hUC-MSCs at various passages (P3, P6, P15) were lysed by TRIzol reagent (ThermoFisher) for total RNA collection according to the manufacturer’s instruction. cDNA was synthesized by using TransScript Fly First-Strand cDNA Synthesis SuperMix (Transgen Biotech, China), and qRT-PCR was performed with the SYBR Green PCR Master Mix (Qiagen) and ABI PRISM 7900 (Applied Biosystems) as we previously reported [25]. The primer sequences are listed in Additional file 7: Table S1.

### Western blotting

Western blotting analysis was conducted as we described before [6, 25]. Briefly, the hUC-MSCs at various passages (P3, P6, P15) were lysed with Laemmli sample buffer (BioRad) and inactivated in 100 °C for 5 min. Then, the samples were electrophoresed in SDS-PAGE gel and transferred onto a PVDF membrane (Life Sciences). After blocking in 5% nonfat milk (BD) for 1 h, the membrane was incubated with primary antibody (Cell Signaling, Abcam) and HRP-conjugated secondary antibody (GE Healthcare). Finally, the membrane was incubated with ECL Detection Reagent (ThermoFisher) and transferred into Super-signal West Pico Chemiluminescent Substrate (Prierce) for development. The antibodies were listed in Additional file 7: Table S3.

### Tri-lineage differentiation analysis of hUC-MSCs

hUC-MSCs at various passages (P3, P6, P15) were seeded at a density of 2 × 10^4^/cm^2^ in MSC culture medium. When cells reached 80% fusion, the medium was changed into adipogenic (MesenCult Adipogenic Differentiation Kit, Stem Cell Technologies), osteogenic (MesenCult Osteogenic Differentiation Kit, Stem Cell Technologies), or chondrogenic (MesenCult-ACF Chondrogenic Differentiation Kit, Stem Cell Technologies) differentiation medium. The differentiation medium was changed every 3 days as we described previously [6, 20]. Twenty-one days later, the hUC-MSC-derived cells were stained by Oil Red S, Alizarin Red, or Alcian Blue staining and photographed with Nikon ElipseTi-U microscope (Nikon, Tokyo, Japan). The primer sequences for tri-lineage differentiation are available in Additional file 7: Table S1.

### Measurement of the secreted cytokines

The contents of the secreted cytokines including IL-6, IL-8, VEGF, G-CSF, HGF, TGF-β1, TNF-α, and PGE-2 in the supernatant were quantified using the enzyme-linked immunosorbent assay (ELISA) kits (R&D Systems) according to the manufactures’ instructions as we previously did [26]. The ELISA kits for the measurement of the secreted cytokines are available in Additional file 7: Table S2.

### Karyotype analysis

The chromosome karyotype analysis was performed to monitor the genomic stability of hUC-MSCs at various passages (P3, P6, P15) using a G-banding technique as we previously reported [5]. Then, the hUC-MSCs (P3, P6, P15) in metaphase were captured by an Olympus DA71 microscope (Tokyo, Japan) with × 200 magnification, and the karyotype was analyzed from the metaphase as we recently reported [5].

### Specimen preparation and whole-exome sequencing

hUC-MSCs at various passages (P3, P6, P15) were used to prepare the whole-exome sequencing samples. For total RNA preparation, the cultured hUC-MSCs (in MSC culture medium) were washed with 1× PBS twice and then lysed by using TRIzol (ThermoFisher) according to the manufacturer’s instructions as we recently reported [6]. For genomic DNA preparation, the hUC-MSCs at indicated passages (P3, P6, P15) were extracted by using a genomic DNA isolation kit (Biovision) according to the manufacturer’s instructions. The RNA and DNA were quantified by using NanoDrop 2000 (ThermoFisher) and sequenced by Novogene (Tianjin, China). The whole-exome sequencing data are available in Additional file 6: Whole Exome Sequencing Data.

### Inhibition of lymphocyte proliferation by coculturing with hUC-MSCs

Human peripheral blood mononuclear cells (PBMCs) were isolated from the blood of healthy donors by standard Ficoll density gradient centrifugation as we previously reported [3]. hUC-MSCs at various passages (P2, P5, P14) were irradiated (by ^137^Cs, 30 Gy) when the cells reached 80% confluency. Then, the irradiated hUC-MSCs were dissociated with 0.25% Trypsin-EDTA at 37 °C for 5 min and resuspended by hUC-MSC culture medium. Then, the prepared 2 × 10^5^ hUC-MSCs (P3, P6, P15) were cocultured with 1 × 10^6^ PBMCs at a ratio of 1:5 in IMDM basal medium (Hyclone) supplemented with 10% FBS and 5 ng/μl PHA addition in 37 °C and 5% CO_2_ incubator. The 1 × 10^6^ PBMCs without hUC-MSC coculture served as a positive control (with 5 ng/μl PHA addition). Seventy-two hours later, the total PBMCs in the supernatant were collected and counted based on trypan blue staining (Procell). Then, the total PBMCs were resuspended in 1× PBS (with 0.2% BSA) and labeled with the indicated antibodies and analyzed by FACS Canto II (BD Biosciences) and FlowJo 7.0 (Ashland). The Th1 cells were labeled with antibodies against CD4 and IFN-γ, the Th2 cells were labeled with antibodies against CD4 and IL-4, the Th17 cells were labeled with antibodies against CD4 and IL17, and the Treg cells were labeled with antibodies against CD4, CD25, and CD127. The antibodies are listed in Additional file 7: Table S3.

### Hematopoietic colony-forming unit assay

Hematopoietic colony-forming unit (CFU) assay was performed as we previously reported with modifications [5, 26]. Briefly, human umbilical blood mononuclear cells (UBMCs) were isolated from the blood of healthy donors by density gradient centrifugation with the Ficoll reagent (Sigma-Aldrich) as we recently reported [26]. The isolated UBMCs were used for CD34^+^ HSC enrichment by using magnetic cell sorting. The enriched CD34^+^ HSCs were then cocultured with hUC-MSCs at various passages (P3, P6, P15) for 14 days in IMDM basal medium (Hyclone) supplemented with 10% FBS in 37 °C and 5% CO_2_ incubator. Then, HSCs in the supernatant were collected and seeded in methylcellulose semi-solid medium (StemCell Tech) as we reported [5]. Finally, the number of hematopoietic CFUs (e.g., CFU-GM) was counted according to the manufacturer’s instructions, and each group’s experiments were implemented for three independent replicates. The morphology of the CFUs is also available in Additional file 3: Figure S3e.

### GVHD mouse model and MSC transplantation

To induce acute GVHD mouse model, the recipient BALB/c mice received a single dose of 10.0 Gy (^60^Coγ) total body irradiation (TBI). Then, the recipient BALB/c mice were injected 1 × 10^7^ spleen cells and 1 × 10^7^ bone marrow cells from the donor C57BL/6 mice as previously reported with several modifications [19, 27, 28]. The irradiated mice were randomly divided into the control group (GVHD) with 1× PBS injection and the experimental groups (GVHD+P3, GVHD+P6, GVHD+P15) with hUC-MSC injection via the tail vein (2 × 10^5^ cells for each mouse at day 6 of the model). Over the next 4 weeks, the clinical and histologic analyses were conducted and recorded as described below.

### Clinical and histologic scoring

Clinical and histologic scoring of mice was monitored as previously reported [19, 27]. The survival, body weight, and appearance of mice in each group were assessed before sacrifice at the indicated time point. The clinical score of GVHD was evaluated using a clinical scoring system as shown in Additional file 4: Figure S4a. For histologic scoring of mice, the sections of the liver, lung, and skin were stained with hematoxylin-eosin (H&E) staining and observed under a Nikon ElipseTi-U microscope (Nikon, Tokyo, Japan) as we described before [26]. Each pathological index of the lung, liver, or skin was graded from 0 to 3, and the total GVHD histologic index was the sum of the parameters listed in Additional file 5: Table S1.

### Histologic section and H&E staining

On day 21 of the acute graft-versus-host disease (aGVHD) model, the liver, lung, and skin samples from euthanized mice of each group were fixed with 10% formaldehyde and embedded in paraffin as we described previously [5, 26]. Then, the fixed samples were made into slides and stained with hematoxylin-eosin (H&E) for photographing and pathological analysis.

### Statistical analysis

Statistical analysis in this study was performed by Prism 6.0 software (GraphPad, San Diego, USA) as we previously reported [6, 25, 26, 29]. Briefly, we used unpaired *t* test to analyze the data of two unpaired groups, while one-way ANOVA with Tukey’s post hoc test was used for the data of multiple unpaired groups. Data were shown as mean ± SEM; *P* < 0.05 were considered statistically significant (*P < 0.05, **P < 0.01, ***P < 0.001; NS, not significant).

## Results

### hUC-MSCs at various passages show similarities in signatures except for proliferation in vitro

Recently, we and other investigators found the therapeutic effect of MSCs was closely related to cell viability and homing capacity, which was fundamental for clinical application and regenerative medicine [5, 22]. Among the influencing factors, long-term in vitro passages would lead to the reduction of the abovementioned characteristics, together with the changes of biological properties and functions, such as the cytokine secretion, hematopoietic supporting and immunomodulating capacities, and even molecular biological alterations [1, 2, 9]. However, systematic study of series in vitro passages on hUC-MSC phenotypes, genotypes, and therapeutic effect is still lacking.

**Fig. 1:**
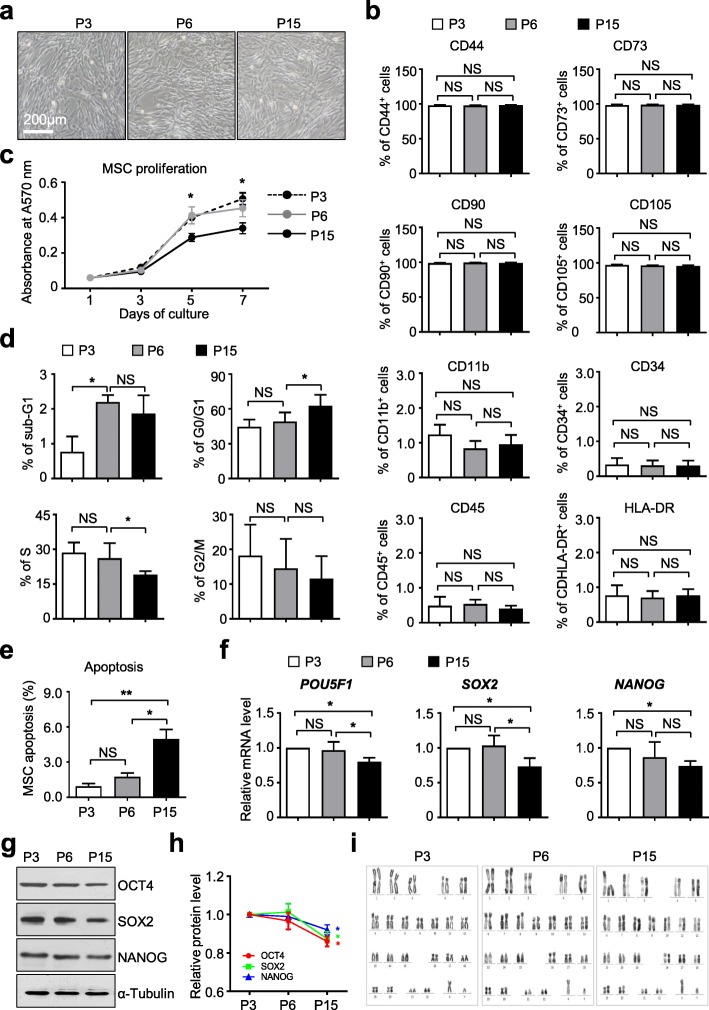
The phenotypic characterization of hUC-MSCs at various passages. **a** Phase contrast images of hUC-MSCs at various passages (P3, P6, P15) in DMEM/F12 medium containing 10% FBS, 1% l-glutamine, 1% NEAA, and 1% penicillin-streptomycin (short for 10% FBS/DF12 medium thereafter). Scale bar = 200 μm. **b** Flow cytometry (FCM) analysis of MSC markers of hUC-MSCs at various passages (P3, P6, P15) cultured in 10% FBS/DF12 medium (mean ± SEM, *n* = 3). NS, not significant. **c** Proliferation assay of hUC-MSCs at various passages (P3, P6, P15) cultured in 10% FBS/DF12 medium for 7 days (mean ± SEM, *n* = 3). *P < 0.05. Cell cycle (**d**) and cell apoptosis (**e**) analysis of hUC-MSCs at various passages (P3, P6, P15) cultured in 10% FBS/DF12 medium by flow cytometry (mean ± SEM, *n* = 3). *P < 0.05; **P < 0.01; NS, not significant. **f** qRT-PCR analysis of pluripotency markers (*POU5F1*, *SOX2*, *NANOG*) in hUC-MSCs at various passages (P3, P6, P15) cultured in 10% FBS/DF12 medium. Data are shown as mean ± SEM (*n* = 3). *P < 0.05; NS, not significant. **g** Western blotting analysis of pluripotency markers (OCT4, SOX2, NANOG) in hUC-MSCs at various passages (P3, P6, P15) cultured in 10% FBS/DF12 medium. α-Tubulin was used as a loading control. **h** Quantitative analysis of OCT4, SOX2, and NANOG expression in hUC-MSCs (P3, P6, P15) cultured in 10% FBS/DMEM-F12 medium by gray scanning with ImageJ software (NIH). Data are shown as mean ± SEM (*n* = 3). *P < 0.05. **i** Karyotype analysis of hUC-MSCs at various passages (P3, P6, P15) cultured in 10% FBS/DF12 medium with G-banded chromosome experiment

For the purpose, we utilized hUC-MSCs at P3, P6, and P15 for subsequent studies. The hUC-MSCs at P2, P5, and P14 were simultaneously thawed and cultured in the abovementioned MSC culture medium. Then, the hUC-MSCs were passaged to P3, P6, and P15. After 3 days, the hUC-MSCs consistently showed typical elongated and spindle-shaped morphologies in culture (Fig. 1a) and without distinguished morphological changes among them. Immunophenotypic identification by flow cytometry analysis showed that hUC-MSCs at the abovementioned passages expressed a high level of mesenchymal surface markers (CD44, CD73, CD90, CD105), together with minimal expression of hematopoietic associated markers (CD11b, CD34, CD45) and HLA-DR (Fig. 1b, Additional file 1: Figure S1a-S1b). Then, by conducting MTT proliferative assay, we found the growth curve of hUC-MSCs at P15 was significantly lower than the other two groups, which indicated the declined proliferative capacity of hUC-MSCs at P15 (Fig. 1c). Consistently, compared to the hUC-MSCs at P15 group, we found more cells were distributed in the sub-S and sub-G2/M phases of the cell cycle, which indicated a higher percentage of cells was in the mitotic period in hUC-MSCs at P3 and P6. By contrast, we also noticed an increasing number of apoptotic cells in hUC-MSCs at P15 (Fig. 1d, e; Additional file 1: Figure S1c). Meanwhile, quantitative analysis of pluripotency markers among hUC-MSCs at various passages showed that the expression of *POU5F1*, *SOX2*, and *NANOG* were decreased slightly in hUC-MSCs at P15, which were further confirmed at the protein level (Fig. 1f–h, Additional file 1: Figure S1d). Furthermore, at the genomic level, by performing a G-banded chromosome experiment, we confirmed the hUC-MSCs at various passages uniformly exhibited normal karyotype without gross abnormalities (Fig. 1i).

### hUC-MSCs at higher passage show difference with those at lower passages in tri-lineage differentiation and cytokine secretion

**Fig. 2:**
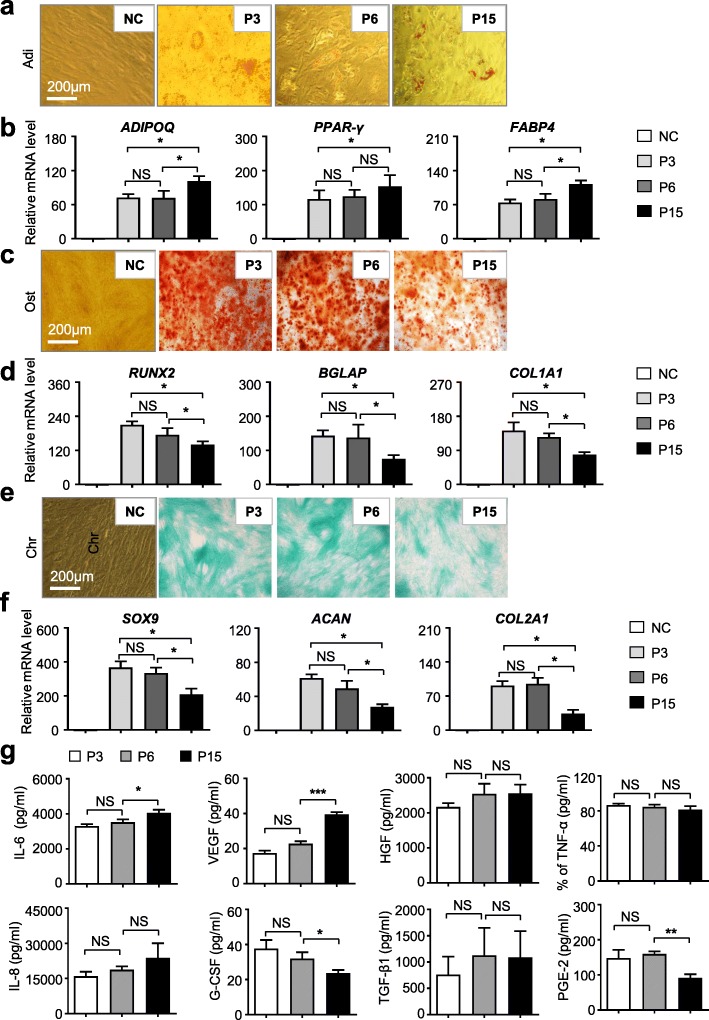
The tri-lineage differentiation potential and cytokine secretion of hUC-MSCs at various passages. **a** Adipogenic differentiation potential of hUC-MSCs at various passages (P3, P6, P15) was identified by Oil Red O staining (scale bar = 200 μm). **b** qRT-PCR analysis of the adipogenic markers (*ADIPOQ*, *PPAR-γ*, *FABP4*) in hUC-MSCs at various passages (P3, P6, P15). Data are shown as mean ± SEM (*n* = 3). *P < 0.05; NS, not significant. All values are normalized to the negative control (NC) group (= 1). **c** Osteogenic differentiation potential of hUC-MSCs at various passages (P3, P6, P15) was identified by Alizarin Red staining (scale bar = 200 μm). **d** qRT-PCR analysis of the osteogenic markers (*RUNX2*, *BGLAP*, *COL2A1*) in hUC-MSCs at various passages (P3, P6, P15). Data are shown as mean ± SEM (*n* = 3). *P < 0.05; NS, not significant. All values are normalized to the NC group (= 1). **e** Chondrogenic differentiation potential of hUC-MSCs at various passages (P3, P6, P15) was identified by Alcian Blue staining (scale bar = 200 μm). **f** qRT-PCR analysis of the chondrogenic markers (*ACAN*, *SOX9*, *COL2A1*) in hUC-MSCs at various passages (P3, P6, P15). Data are shown as mean ± SEM (*n* = 3). *P < 0.05; NS, not significant. All values are normalized to the NC group (= 1). **g** ELISA shows soluble cytokines (IL-6, IL-8, VEGF, G-CSF, HGF, TGF-β, TNF-α, PGE-2) in the supernatant of hUC-MSCs at various passages (P3, P6, P15) after 24 h culture in MSC medium. All values normalized to level (= 1) of cytokine concentration in P3. Data shown as mean ± SEM (*n* = 3). *P < 0.05; ***P < 0.001; NS, not significant.

To clarify the potential influence of long-term in vitro passages on the differentiation properties of hUC-MSCs, we initially conducted multi-lineage differentiation analyses as we previously reported [5, 6, 20]. Oil Red staining showed that there were more lipid droplets with high refractivity in hUC-MSCs at P15 than those in hUC-MSCs at P3 or P6 (Fig. 2a) after 3 weeks of adipogenic differentiation. With the aid of quantitative analysis of adipogenic markers, we found higher levels of *ADIPOQ*, *PPAR-γ*, and *FABP4*, were expressed in the P15 group (Fig. 2b). Conversely, the osteogenic differentiation potential of hUC-MSCs at P15 was weaker than that in the P3 and P6 groups, which was confirmed by both Alizarin Red staining and qRT-PCR analysis of osteogenic markers, *RUNX2*, *BGLAP*, and *COL1A1* (Fig. 2c, d). Similarly, the chondrogenic differentiation potential of hUC-MSCs at P15 was weaker than that of hUC-MSCs at P3 and P6 as well, which was confirmed by both Alcian Blue staining and qRT-PCR analysis of chondrogenic markers, *SOX9*, *ACAN*, and *COL2A1* (Fig. 2e, f). Meanwhile, we did not find significant differences between hUC-MSCs at P3 and P6 in multi-lineage differentiation capacity.

Furthermore, we were curious about whether the capacity of cytokine secretions could also be affected by a series of long-term in vitro culture and passages. For the purpose, we cultured hUC-MSCs at P3, P6, and P15 for 24 h, then the supernatants were immediately collected after a rapid centrifugation for the indicated cytokine detection. Compared to the other groups, hUC-MSCs at P15 secreted more IL-6 and VEGF but less G-CSF and PGE-2 as well. In addition, there were no significant differences in other cytokine secretions among the hUC-MSCs at various passages, such as IL-8, HGF, TGF-β1, and TNF-α (Fig. 2g). Taken together, these data demonstrated that hUC-MSCs at higher passages could affect their signature such as multi-lineage differentiation and cytokine secretion potential.

### hUC-MSCs at various passages have multiple mutation spectrum but without tumor formation capacity in vivo

**Fig. 3:**
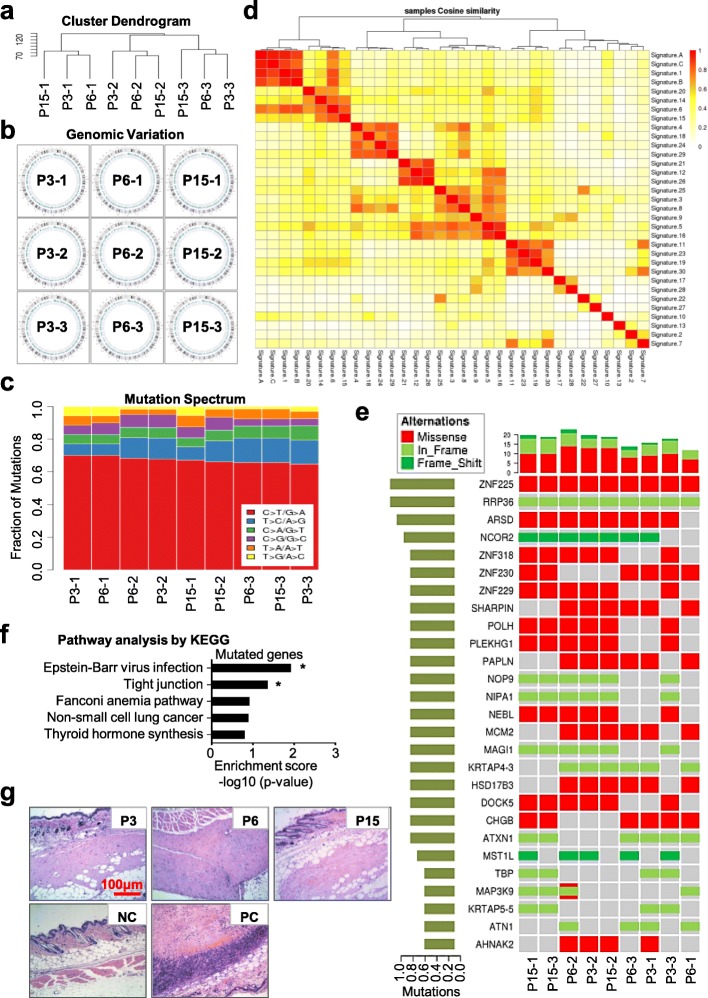
Safety evaluation of hUC-MSCs at various passages by mutation spectrum and tumor formation analysis. **a** Hierarchical clustering analysis of hUC-MSCs at various passages (P3, P6, P15) based on SNP data of 334,378 exons. **b** Cumulative somatic mutation analysis of genetic mutations in hUC-MSCs at various passages (P3, P6, P15) by Circos software. **c** Fraction of high-frequency SNV mutations among the indicated hUC-MSCs at various passages (P3, P6, P15). **d** The nonnegative matrix factorization algorithm (NMF) showed that the point mutations were refined into 96 subsets based on the location of 1 bp base around the upstream and downstream. Then, unsupervised hierarchical clustering analysis of the different signatures (signature A, B, C) with the 30 kinds of characteristics of somatic point mutations. **e** High-frequency mutation analysis of hUC-MSCs at various passages (P3, P6, P15) including single nucleotide polymorphism (SNP) and INDEL mutations by using the MuSic test. **f** Metabolic pathway enrichment analysis of the genes with high-frequency mutations in hUC-MSCs at various passages (P3, P6, P15) by using PathScan software. **g** Tumorigenicity assay of hUC-MSCs at various passages (P3, P6, P15) by subcutaneous injection of the 1.0 × 10^7^ hUC-MSCs mixed with Matrigel into nude mice. Mice with Matrigel injection were used as the negative control (NC), mice with 1.0 × 10^6^ Hela cells mixed with Matrigel injection were used as the positive control (PC). H&E staining was performed for tumor structure detection (scale bar = 100 μm)

To further estimate the potential influence of serial passages and long-term in vitro culture to the chromosome karyotype stability at the molecular level of hUC-MSCs, we took advantages of the Illumina Hiseq platform for whole-exosome sequencing of three independent hUC-MSC cell lineages at P3, P6, and P15. Initially, unsupervised hierarchical clustering analysis based on the SNP data of 334,378 exons showed that the hUC-MSCs at various passages with the same origin had much more similarities in global SNP signature (Fig. 3a, Additional file 2: Figure S2a-2b). The somatic variation analysis by the Circos software further conformed the clustering result (Fig. 3b). Furthermore, the fractions of single nucleotide variation (SNV) including mutation spectrum and mutation signature were further calculated. As shown by the chart and heatmap diagrams, there were 6 types of SNV mutations, including C>A/G>T, C>G/G>C, C>T/G>A, T>A/A>T, T>C/A>G, and T>G/A>C. In general, hUC-MSCs at various passages with the same origin (e.g., P3-1, P6-1, and P15-1) showed a more similar pattern of the point mutation spectrum compared to those of the hUC-MSCs with different origins (Fig. 3c, Additional file 2: Figure S2c). We also noticed that the major type of SNV mutations was C>T/G>A and the proportion of this mutation was distinguishable among all the abovementioned hUC-MSCs, while significant differences of T>C/A>G and C>G/G>C mutations were visible as well, which were confirmed by the heatmap diagram of clustering analysis (Fig. 3c, Additional file 2: Figure S2c). Furthermore, the point mutations were further refined into 96 subsets based on the location of 1 bp base around the upstream and downstream, and then 30 kinds of characteristics of somatic point mutations were defined according to the common nonnegative matrix factorization algorithm (NMF) as shown in the COSMIC website (Fig. 3d, Additional file 2: Figure S2d). Not surprisingly, hUC-MSCs at various passages with the same origin (from the first person—P3-1, P6-1, P15-1; from the second person—P3-2, P6-2, P15-2; or from the third person—P3-3, P6-3, P15-3) shown the same signature of somatic point mutations (e.g., signature A, B, or C), which indicated the genomic stability. However, hUC-MSCs at P3, P6, and P15 with different origins (e.g., P3-1, P6-2, P15-3 from the abovementioned 3 independent persons, respectively) exhibited diversity in the signature of somatic point mutations (Fig. 3d, Additional file 2: Figure S2d-2e).

Next, to evaluate the potential correlation with risk of tumorigenesis, we conducted a high-frequency mutation analysis based on the significantly mutated genes (SMG) including single nucleotide polymorphism (SNP) and INDEL mutations by using the MuSic test. From the personalized display of cancer somatic cell panorama mutations, we could intuitively observe the mutation rate, high-frequency mutant genes, and mutation types (Fig. 3e). For instance, we found a number of genes with the same missense (e.g., *ZNF225*) or frameshift (e.g., *RRP36*), while most of the listed genes with variations even with the same origin such as *ZNF318*, *NCOR2*, and *DOCK5* (Fig. 3e). Metabolic pathway enrichment analysis by using PathScan software showed that the enriched genes with high-frequency mutations were significantly relative to Epstein-Barr virus infection or tight junction, while no statistical significance on the Fanconi anemia pathway, non-small cell lung cancer, or throid hormone synthesis (Fig. 3f, Additional file 3: Figure S3a). Additionally, we also checked the mutation of 11 key tumor-initiating genes (*MYC*, *BMI1*, *HRAS*, *MYB*, *FOS*, *MET*, *JUN*, *EGFR*, *CSF1R*, *ZHX2*, and *ABL1*), together with 10 vital tumor-inhibiting genes (*P53*, *Pb*, *P16*, *P21*, *APC*, *DCC*, *NF1*, *NF2*, *VHL*, and *WT1*) as well; collectively, we did not find new harmful SNP mutations in the abovementioned 21 genes (Additional file 6: Whole Exome Sequencing Data). Hence, although with mutation spectrum during long-term in vitro culture and passages, the genetics of hUC-MSCs at various passages including P3, P6, and P15 were stable and safe.

Finally, to further evaluate the safety of MSCs in vivo, we conducted a subcutaneous tumorigenesis test and found there were no tumor structures formed by hUC-MSCs at various passages (Fig. 3g). Simultaneously, we transplanted the abovementioned hUC-MSCs into NOD/SCID mice by tail intravenous injection to analyze the distribution and colonization of hUC-MSCs in vivo. With the aid of fluorescent quantitative real-time PCR (FQ-RT-PCR), we calculated the content of hUC-MSCs in tissues based on the amplification curve and standard curve (Additional file 3: Figure S3b-3c). Interestingly, we found the transplanted hUC-MSCs at P3, P6, or P15 could be detected in the lung and/or kidney within the first day (day 1), and then the cells could migrate to the femur and heart at day 21 (Additional file 3: Figure S3d).

### hUC-MSCs at lower passages exhibit superiority in immunomodulating and hematopoietic supporting capacities in vitro

**Fig. 4:**
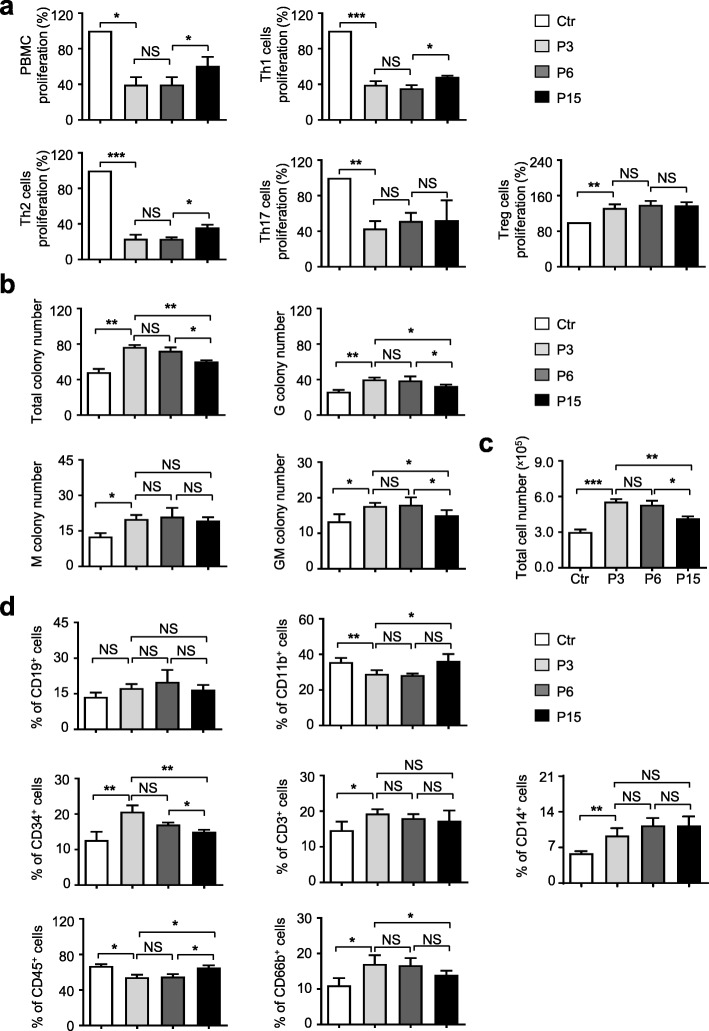
Hematopoietic supporting effect of hUC-MSCs at various passages in vitro. **a** hUC-MSCs at various passages were cultured with PBMC. The inhibition of lymphopoiesis or specific sub-lymphocyte proliferation (Th1, Th2, Treg, Th17) was normalized to the level (= 1) of those in the Ctr group. Data shown as mean ± SEM (*n* = 3). *P < 0.05; **P < 0.01; ***P < 0.001; NS, not significant. **b**, **c** hUC-MSCs at various passages were cultured in 10% FBS/IMDM medium for 72 h; the supernatant was collected and cocultured with PBMCs for 14 days. The total colony number, G colony number, M colony number, GM colony number, and total PBMC number were counted and calculated. **d** Flow cytometry analysis of the cocultured PBMC-derived cell population with indicated antibodies (CD19, CD11b, CD34, CD3, CD14, CD45, CD66b). All values normalized to level (= 1) of those in the Ctr group. Data shown as mean ± SEM (*n* = 3). *P < 0.05; **P < 0.01; ***P < 0.001; NS, not significant

Immunomodulating and hematopoietic supporting capacities have been recognized as the two unique characteristics of adult MSCs [2]. Originally, as described in the “Methods” section, we cocultured PBMCs with the aforementioned irradiated hUC-MSCs (P3, P6, P15) to evaluate the immunomodulating function of hUC-MSCs at the indicated passages. Seventy-two hours later, the total PBMCs and subpopulations including Th1, Th2, Th17, and Treg cells were analyzed to evaluate the inhibitory effect of hUC-MSCs. Compared with the P3 and P6 groups, we found the P15 group showed significantly attenuated capacity of inhibiting PBMCs, which was identified by cell counting. Furthermore, with the aid of flow cytometry analysis, we noticed that the inhibitory effect of hUC-MSCs at P15 on Th1 and Th2 cell proliferation was decreased, while hUC-MSCs at various passages showed indistinguishable functions on Treg and Th17 cells proliferation (Fig. 4a).

Then, to compare the hematopoietic supporting capacity among the hUC-MSCs at P3, P6, and P15, we conducted the traditional hematopoietic colony-forming unit (CFU) assay as we previously reported [5]. After 14 days of culture in methylcellulose medium, the UCB CD34^+^ HSC-derived colonies were observed and calculated. When compared with the P3 or P6 group, the numbers of total colonies, G colonies, GM colonies, and even total cells were simultaneously declined in the P15 group (Fig. 4b, c; Additional file 3: Figure S3e). After that, the UCB CD34^+^ HSC-derived hematopoietic CFUs were collected for subpopulation analysis. In a coincidence, a lower percentage of CD34^+^ and CD66^+^ cells and a higher percentage of CD11b^+^ and CD45^+^ cells were emergent in the P15 group, while the proportions of other subpopulations showed no significant differences such as the CD19^+^, CD3^+^, and CD14^+^ subpopulations (Fig. 4d). Taken together, these data indicated that long-term in vitro culture or continuous passages could partially alter the signatures and properties of hUC-MSCs.

### hUC-MSCs at lower passage exhibit therapeutic effect on GVHD mice in vivo

**Fig. 5:**
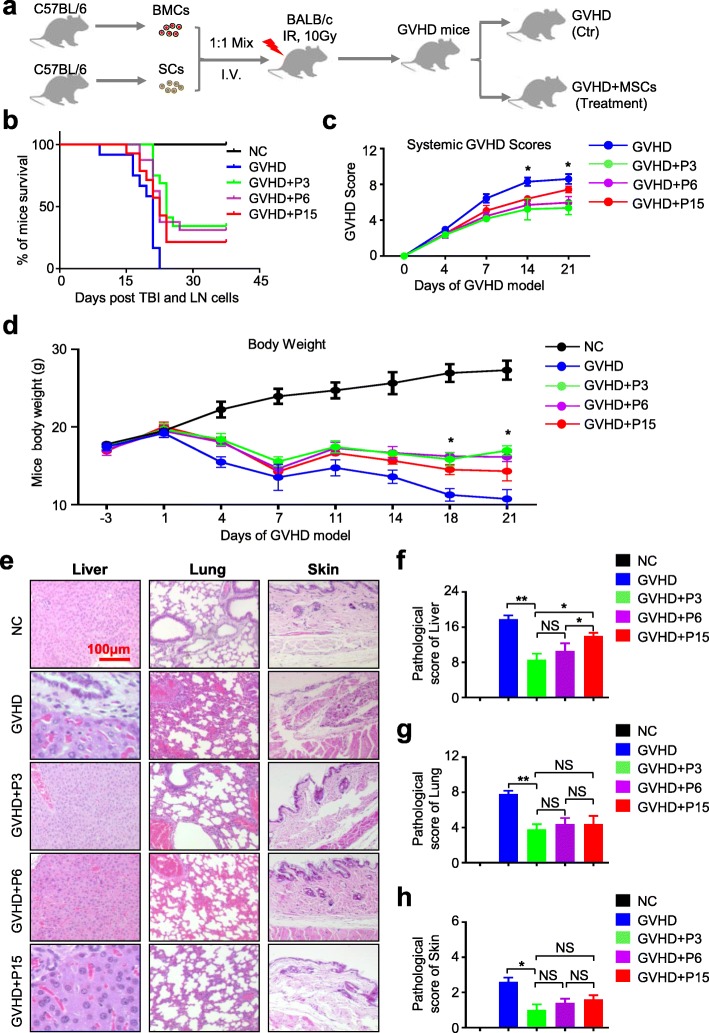
The UC-MSCs at the lower passage exhibit superiority in therapeutic effect on GVHD mice in vivo. **a** Schematic of the establishment of aGVHD mouse model and MSC transplantation. Briefly, bone marrow cells (BMCs) and spleen cells (SCs) were collected from C57BL/6 donor mice, then BMCs and SCs were mixed by 1:1 and transfused into the recipient BALB/c mice with 10 Gy total body irradiation (TBI) treatment via tail intravenous injection under sterile conditions to induce aGVHD. The aGVHD mice were randomly divided into the Ctr group (with 1× PBS injection) and experimental groups (GVHD+MSCs: P3, P6, P15). **b** Overall survival in aGVHD mice given hUC-MSCs at various passages (GVHD+P3, GVHD+P6, GVHD+P15) or not given MSCs (GVHD). **c** GVHD scores of mice in the Ctr group and experimental groups (GVHD+MSCs: P3, P6, P15) following induction of the model. **d** Body weight of mice in the Ctr group and experimental groups (GVHD+MSCs: P3, P6, P15) following induction of the model. **e** Microscopic features of the liver, lung, and skin at the time point of necropsy in the Ctr group and experimental groups (GVHD+MSCs: P3, P6, P15). Scale bar = 100 μm. Pathological scores of the liver (**f**), lung (**g**), and skin (**h**) based on the sections of H&E staining. Data shown as mean ± SEM (*n* = 3). *P < 0.05; **P < 0.01; NS, not significant

For the purpose of further evaluating the similarities and differences of biological function among the abovementioned hUC-MSCs in vivo, we took advantage of the common GVHD model [19, 27, 28]. As shown in Fig. 5a, to induce GVHD model, 1 × 10^7^ bone marrow cells (BMCs) and 1 × 10^7^ spleen cells (SCs) from the donor C57BL/6 mice were mixed and intravenously injected into the recipient BALB/c mice with 10-Gy total body irradiation (TBI) prior to transplantation via the tail vein. After that, the GVHD mice were randomized into the control group (GVHD) with saline injection and the experimental groups with 2 × 10^5^ hUC-MSC (GVHD+P3, GVHD+P6, or GVHD+P15) treatment (Fig. 5a). Compared to the GVHD group, mice received systemic infusion of hUC-MSCs exhibited reduced mortality and prolonged survival, while the therapeutic effect of hUC-MSCs at P15 was weaker than the P3 and P6 groups (Fig. 5b). Consistent with the previous studies, the transplantation of allogeneic BMCs and SCs induced the recipient mice with high GVHD scores and lethality of 100% within 21 days. Different from the GVHD+P15 group, hUC-MSCs at P3 and P6 showed a more significant protective effect on GVHD mice and attenuated the GVHD symptoms, including the low clinical score and body weight decline (Fig. 5c, d; Additional file 4: Figure S3a).

Consistent with the observation of general signs, the GVHD mice with hUC-MSC injection also showed significantly alleviated GVHD-associated pathology in the liver, lung, and skin compared to the GVHD group, particularly the infiltration of inflammatory cells, vacuolization, and tissue necrosis (Fig. 5e). Histological scores of the livers showed that mice with P3 hUC-MSC infusion were comparable to those mice with P6 hUC-MSC injection but better than those mice in the GVHD+P15 group (Fig. 5f, Additional file 5: Table S1). A histological examination of the lungs in the indicated groups showed that hUC-MSC infusion could dramatically decrease lymphocyte infiltration, especially in mice with P3 and P6 hUC-MSC transplantation (Fig. 5g, Additional file 5: Table S1). With regard to the skin, mice in the GVHD group exhibited severe epidermal absence and inflammatory cell infiltration in the sebaceous glands and appendages, while mice in the experimental groups with hUC-MSCs had slight symptoms, such as balloon-like changes of keratinocytes in the basal layers or mild inflammatory cell infiltration (Fig. 5h, Additional file 5: Table S1). Collectively, systemic administration of hUC-MSCs at various passages could significantly prolong the survival of mice with aGVHD and ameliorate the clinical aGVHD symptoms and pathological damages of mice, particularly in the acknowledged aGVHD-targeted organs.

## Discussion

Effective application of stem cells including hUC-MSCs in regenerative medicine relies on the generation of high-quality cell populations with characteristics such as safety, effectiveness, and reproducibility [30]. However, the differentiation trajectories and cellular heterogeneity of in vitro cultured hUC-MSCs remain largely unclear [31, 32]. Herein, in this study, we systematically evaluated the biological signatures, genetic mutation, and unique functions of hUC-MSCs at various passages both at the cellular and molecular levels for the first time. Generally, although there were merely visible differences in morphologic characteristics and immunophenotypes, yet hUC-MSCs at the higher passage (P15) showed more distinguishable differences in various biological process and whole exome from the lower passages (P3, P6). Taken together, our studies provided indispensable new references for standardization of hUC-MSCs for clinical application.

Since the 1960s, MSCs were firstly separated and cultured in vitro [8]. After that, we and other investigators focused on the paramount potential of MSCs in disease treatment and tissue repair [5, 6, 15, 19, 24]. Indeed, MSCs with different origins were wildly used in numerous disease treatment, such as hematological malignancies, acute-on-chronic liver failure (ACLF), osteoarthritis (OA), cerebral palsy, type I or II diabetes, and severe hindlimb ischemia [14, 15, 33]. These inspiring studies indicated the enormous potential of MSCs in regenerative medicine [1, 13]. However, several studies also indicated that the therapeutic effects of MSCs were not convenient and even controversial to some extent [12, 16, 18, 34]. Most of all, before preclinical or clinical applications, the standardization of MSCs by systematic evaluation was almost lacking, which would be a foremost important problem for cell vitality and homing, even for recipients’ safety [4, 9, 31, 35]. To date, although BM-MSCs act as the most widely used cells in clinical trials, yet hUC-MSCs possess superiority in large-scale expansion and standardization [31, 36]. Of the listed factors, a number of studies indicated that long-term in vitro culture and continuous passages were the prohibiting factor for phenotype and function of MSCs, yet the evidence was still lacking [21, 22, 31]. Currently, even though we and other investigators have compared the biological signatures of hUC-MSCs with hBM-MSCs or hAD-MSCs in some respects at the cellular level, few of the studies are further focusing on the distinctions at the molecular level [21, 37]. Very recently, by utilizing single-cell transcriptomic analysis, Huang et al. reported that the in vitro expanded hUC-MSCs showed heterogeneity in inflammatory cytokines and cell cycle stages [31], which was consistent with our data by whole-exome sequencing. In addition, cell cycle trajectory has a high positive correlation with the heterogeneity as well [31].

The potential influence of long-term in vitro passages on the hereditary stability of MSCs including hUC-MSCs is also largely unknown. In this study, with the aid of whole-exome sequencing, we found hUC-MSCs at various passages or from independent individuals have limited SNV and INDEL mutations in multiple features, which delineated the high conservativeness of variability among the whole-exome datasets. Meanwhile, a recent study found that culture condition could simultaneously influence the transcriptome and proteome expression profiles of MSCs [32]. Thus, there is a suspicious attitude towards the potential risk of continuous passages on MSCs in considering the allogeneic cell sources and the probability of genetic variability. Herein, with the aid of high-frequency mutation analysis and metabolic pathway enrichment analysis, we consistently observed the similar phenomenon that the mutations did not show correlation with the risk of tumorigenesis as well [31]. However, as to specific SNV and INDEL mutation calculation, there were indeed differences in alternation among the abovementioned hUC-MSCs. Together with the in vivo transplantation analysis, our studies provided overwhelming evidence for the safety of hUC-MSCs after continuous in vitro passages both at the cellular and molecular levels for the first time. Above all, we systematically analyzed the biological signatures, safety, genetic mutation pattern, and the recommended function of hUC-MSCs for the first time. Our studies would supply new references and benefit the basic research and regenerative medicine in future.

## Conclusions

Overall, in this study, we systematically evaluated the biological signatures, in vitro and in vivo functions, and mutation pattern of hUC-MSCs at various passages. Our studies would supply references for the fundamental research and clinical applications in future.

## Supplementary information

**Additional file 1: Figure S1.** Identification of UC-MSCs at various passages by flow cytometry.

**Additional file 2: Figure S2.** Identification of mutation spectrum and content of hUC-MSCs at various passages.

**Additional file 3: Figure S3.** Identification of tissue distribution and hematopoietic-supporting effect of hUC-MSCs.

**Additional file 4: Figure S4.** Clinical symptoms and physical index scores of aGVHD mice.

**Additional file 5: Table S1.** The pathological index scores of aGVHD mice including the liver, lung and skin.

**Additional file 6.** The whole exome sequencing data of hUC-MSCs (P3, P6, P15) from three individuals were listed.

**Additional file 7.** The details accompanied with the main manuscript including Additional Figure Legends and Additional Tables were listed.

## Data Availability

All data generated or analyzed during this study are included in this published article and its supplementary information files. Meanwhile, the datasets used and analyzed during the current study are also available from the corresponding author on reasonable request.

## Abbreviations

ACLF: Acute-on-chronic liver failure
BFU-E: Burst-forming unit erythroid
CFU-E: Colony-forming unit erythrocyte
CFU-GEMM: Colony-forming unit granulocyte/erythroid/macrophage/megakaryocyte
CFU-GM: Colony-forming unit granulocyte/macrophage
ELISA: Enzyme-linked immunosorbent assay
H&E: Hematoxylin-eosin
MLC: Mixed lymphocyte cultivate
PBMCs: Peripheral blood mononuclear cells
SNP: Single nucleotide polymorphism
SNV: Single nucleotide variation
UC-MSCs: Umbilical cord-derived MSCs
SMG: Significantly mutated genes
hPSCs: Human pluripotent stem cells
RA: Rheumatoid arthritis
CLI: Critical limb ischemia
