# Supplementary figures and images for: Systematic comparison of hUC-MSCs at various passages reveals the variations of signatures and therapeutic effect on acute graft-versus-host disease
Source: Stem Cell Res Ther. 2019 Nov 28;10:354. doi: 10.1186/s13287-019-1478-4 (PMC6883552; doi:10.1186/s13287-019-1478-4)

**a**

| UC-MSCs | CD44  | CD73  | CD90  | CD105 | CD11b | CD34 | CD45 | HLA-DR |
|---------|-------|-------|-------|-------|-------|------|------|--------|
| P3-1    | 97.82 | 99.06 | 98.23 | 95.01 | 1.96  | 0.10 | 0.16 | 0.35   |
| P3-2    | 98.3  | 98.84 | 99.70 | 96.33 | 0.66  | 0.18 | 0.25 | 0.26   |
| P3-3    | 99.5  | 99.86 | 99.33 | 97.95 | 1.38  | 0.16 | 1.26 | 1.51   |
| P6-1    | 98.1  | 99.06 | 99.39 | 95.90 | 1.09  | 0.18 | 0.66 | 1.13   |
| P6-2    | 96.8  | 98.92 | 99.62 | 95.70 | 0.27  | 0.22 | 0.17 | 0.22   |
| P6-3    | 97.9  | 99.44 | 99.75 | 96.84 | 1.24  | 0.11 | 0.57 | 0.63   |
| P15-1   | 98.3  | 99.06 | 99.39 | 97.95 | 0.24  | 0.13 | 0.18 | 0.28   |
| P15-2   | 99.2  | 99.86 | 99.81 | 95.93 | 1.31  | 0.18 | 0.51 | 1.09   |
| P15-3   | 97.8  | 98.94 | 99.95 | 95.78 | 1.42  | 0.15 | 0.54 | 0.95   |

**b**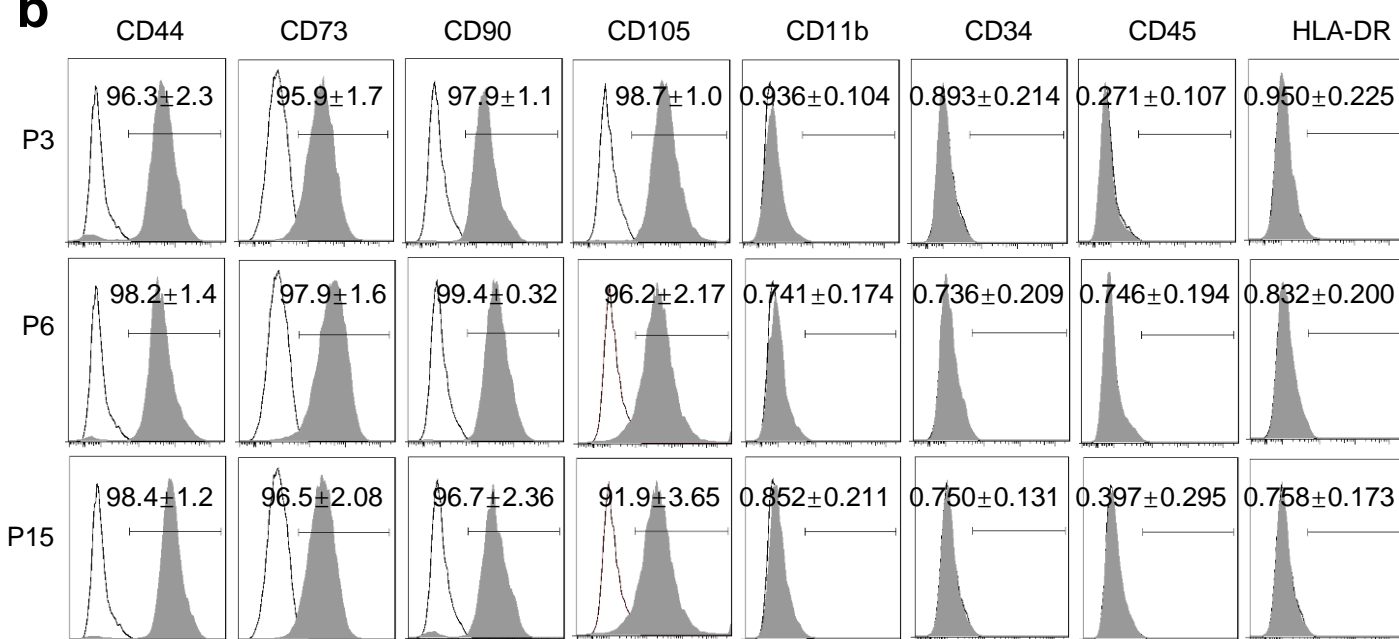**c**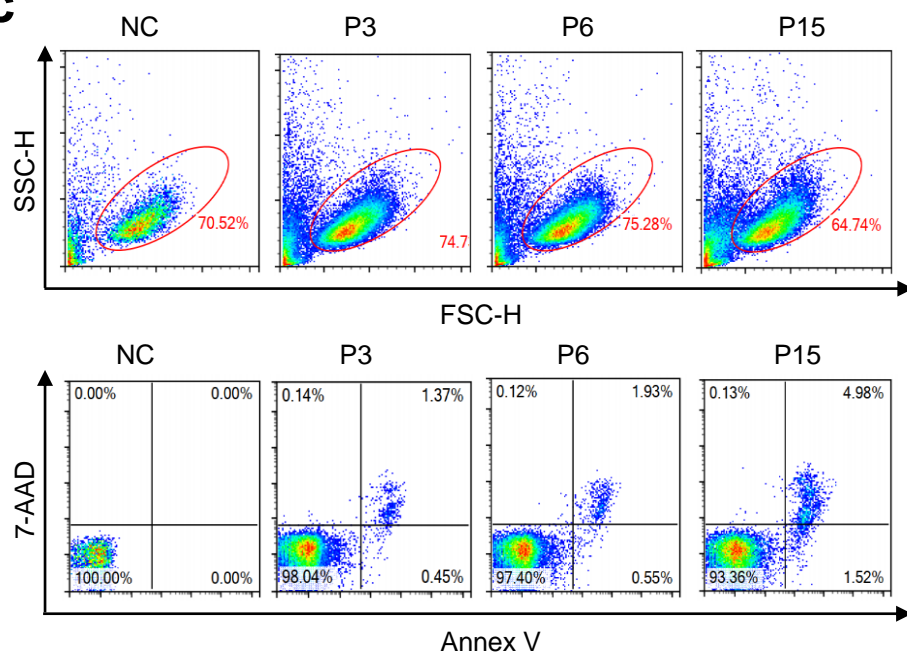**d**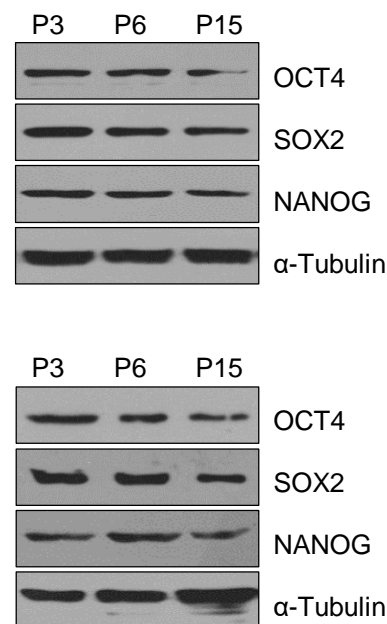

Supplement: Supplementary file 1 — Additional file 1: Figure S1. Identification of UC-MSCs at various passages by flow cytometry. [file 13287_2019_1478_MOESM1_ESM.pdf]

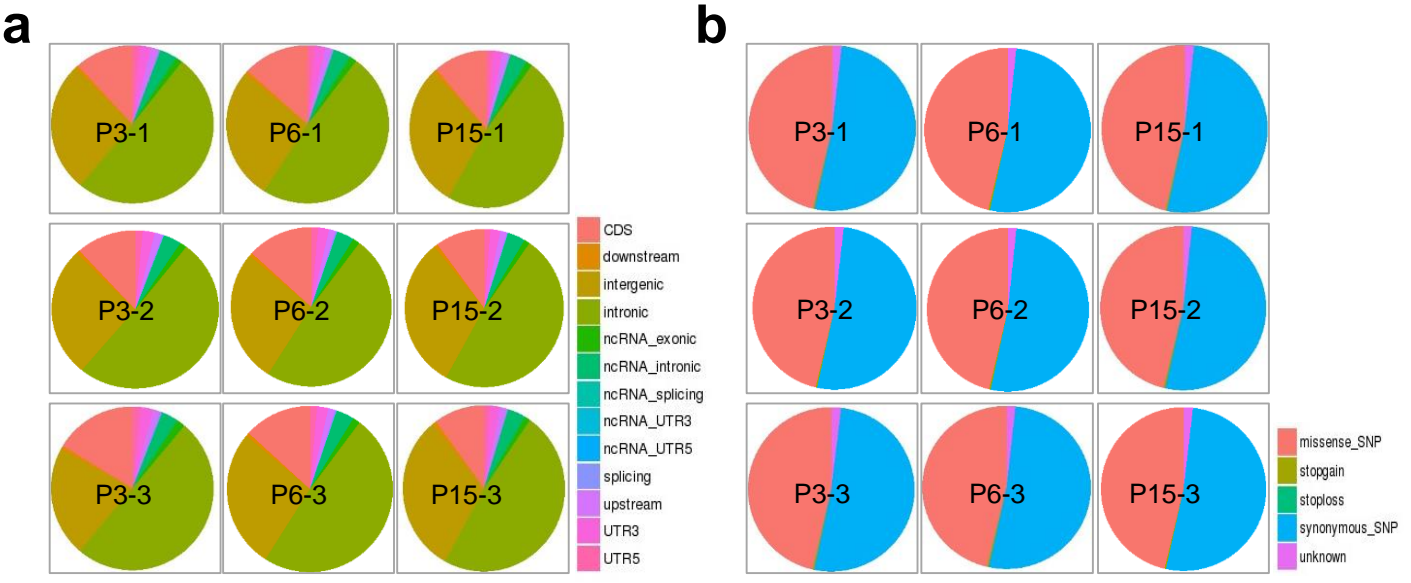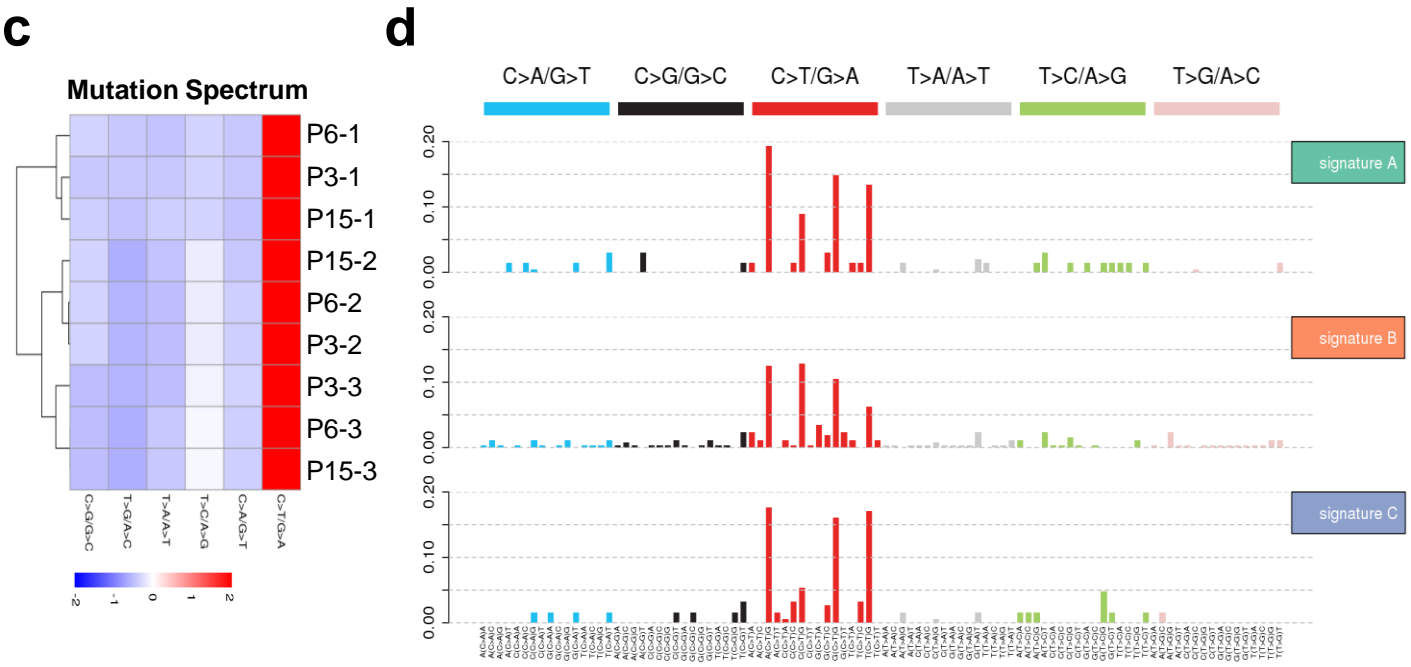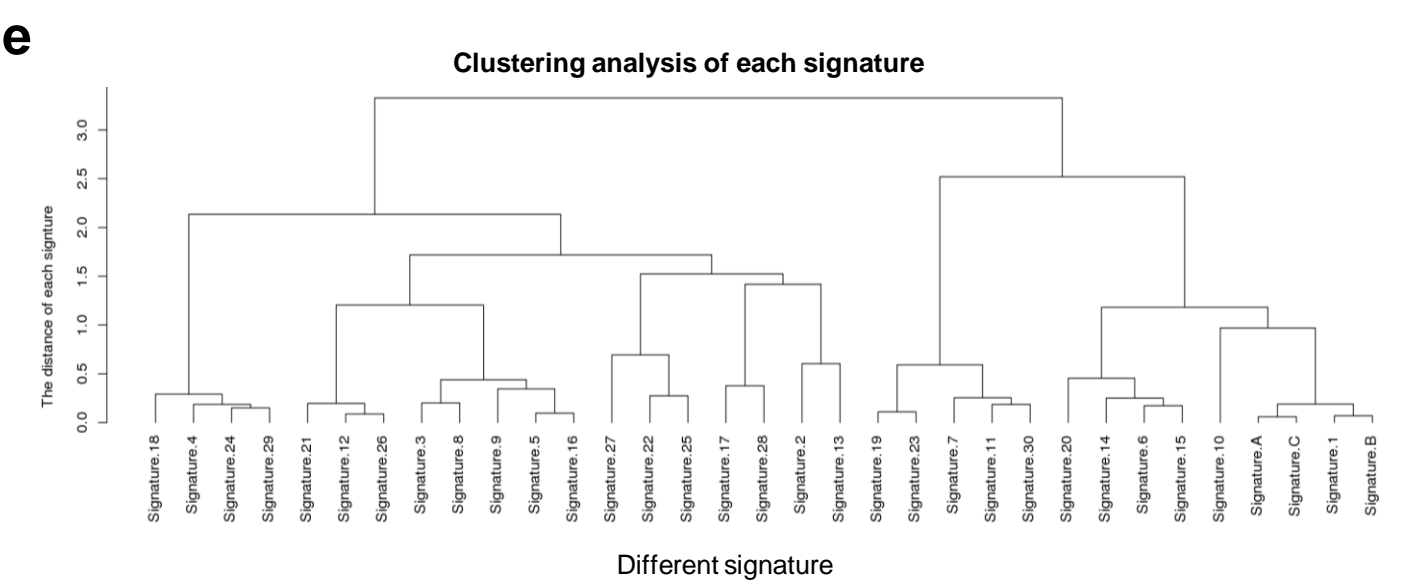

Supplement: Supplementary file 2 — Additional file 2: Figure S2. Identification of mutation spectrum and content of hUC-MSCs at various passages. [file 13287_2019_1478_MOESM2_ESM.pdf]
