# Supplementary material for: Systematic comparison of hUC-MSCs at various passages reveals the variations of signatures and therapeutic effect on acute graft-versus-host disease
Source: Stem Cell Res Ther. 2019 Nov 28;10:354. doi: 10.1186/s13287-019-1478-4 (PMC6883552; doi:10.1186/s13287-019-1478-4)

a

Pathway	Pathway_name	Pathway_id	Database	fg_items	bg_items	rich_factor	pvalue	qvalue	Genes
hsa05205	Proteoglycans in cancer	PROTEOGLYCANS IN CANCER	KEGG	1	205	0	3.97E-01	4.23E-01	TP53
hsa04151	PI3K-Akt signaling pathway	PI3K-AKT SIGNALING PATHWAY	KEGG	2	341	0.01	1.99E-01	2.88E-01	PPP2R5E|T
hsa04210	Apoptosis	APOPTOSIS	KEGG	1	140	0.01	2.91E-01	3.41E-01	TP53
hsa04110	Cell cycle	CELL CYCLE	KEGG	1	124	0.01	2.62E-01	3.34E-01	TP53
hsa05223	Non-small cell lung cancer	NON-SMALL CELL LUNG CANCER	KEGG	1	56	0.02	1.28E-01	2.79E-01	TP53
hsa04530	Tight junction	TIGHT JUNCTION	KEGG	2	139	0.01	4.37E-02	2.79E-01	MPDZ|MA
hsa03015	mRNA surveillance pathway	MRNA SURVEILLANCE PATHWAY	KEGG	1	91	0.01	1.99E-01	2.88E-01	PPP2R5E
hsa04933	AGE-RAGE signaling pathway in diabetic	AGE-RAGE SIGNALING PATHWAY	KEGG	1	101	0.01	2.19E-01	3.03E-01	DIAPH1
hsa03460	Fanconi anemia pathway	FANCONI ANEMIA PATHWAY	KEGG	1	53	0.02	1.21E-01	2.79E-01	REV1
hsa05160	Hepatitis C	HEPATITIS C	KEGG	1	133	0.01	2.78E-01	3.41E-01	TP53
hsa04211	Longevity regulating pathway	LONGEVITY REGULATING PATHW	KEGG	1	94	0.01	2.05E-01	2.90E-01	TP53
hsa05161	Hepatitis B	HEPATITIS B	KEGG	1	146	0.01	3.01E-01	3.41E-01	TP53
hsa05010	Alzheimer's disease	ALZHEIMER'S DISEASE	KEGG	1	168	0.01	3.38E-01	3.73E-01	LRP1
hsa05169	Epstein-Barr virus infection	EPSTEIN-BARR VIRUS INFECTION	KEGG	3	202	0.01	1.19E-02	2.68E-01	TBP|TP53|
hsa05215	Prostate cancer	PROSTATE CANCER	KEGG	1	89	0.01	1.95E-01	2.88E-01	TP53
hsa04918	Thyroid hormone synthesis	THYROID HORMONE SYNTHESIS	KEGG	1	71	0.01	1.59E-01	2.79E-01	TG
hsa05218	Melanoma	MELANOMA	KEGG	1	71	0.01	1.59E-01	2.79E-01	TP53
hsa05222	Small cell lung cancer	SMALL CELL LUNG CANCER	KEGG	1	86	0.01	1.89E-01	2.88E-01	TP53

b

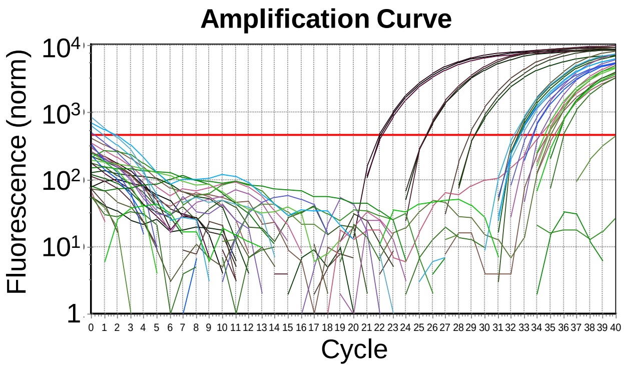

c

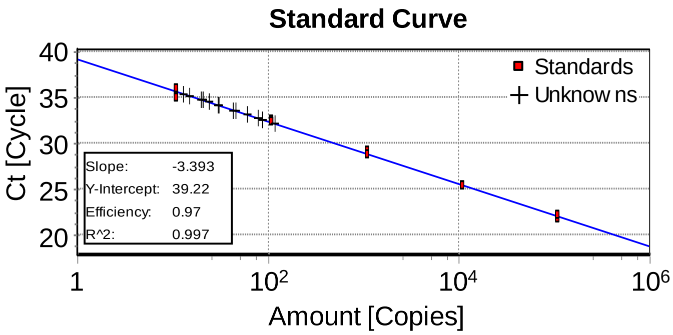

d

Content of hUC-MSCs in tissues

Time (day)	Mice (NO.)	Organ	*β-globin* (copies)	Total DNA Con (μg)	Relative MSC Con (ng/μg)
1	3206	Lung	18.8	0.065	4.32
1	3206	Kidney	11.9	0.500	0.35
1	3207	Lung	94.0	0.415	3.38
1	3208	Lung	21.8	0.500	0.65
1	3209	Lung	32.2	0.500	0.96
1	3209	Femur	25.6	0.500	0.76
1	3210	Lung	62.9	0.500	1.88
21	3212	Heart	12.3	0.500	0.37
21	3215	Heart	68.1	0.500	2.03
21	3215	Femur	40.2	0.500	1.20

e

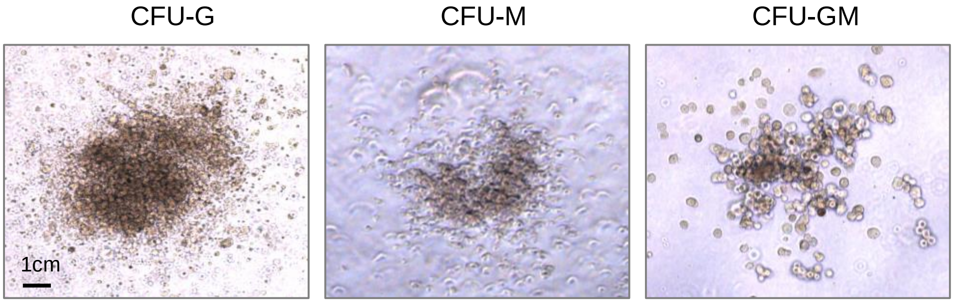

Supplement: Supplementary file 3 — Additional file 3: Figure S3. Identification of tissue distribution and hematopoietic-supporting effect of hUC-MSCs. [file 13287_2019_1478_MOESM3_ESM.pdf]
