# Supplementary material for: Systematic comparison of hUC-MSCs at various passages reveals the variations of signatures and therapeutic effect on acute graft-versus-host disease
Source: Stem Cell Res Ther. 2019 Nov 28;10:354. doi: 10.1186/s13287-019-1478-4 (PMC6883552; doi:10.1186/s13287-019-1478-4)
Supplement: Supplementary file 4 — Additional file 4: Figure S4. Clinical symptoms and physical index scores of aGVHD mice. [file 13287_2019_1478_MOESM4_ESM.pdf]

a

Clinical symptoms and physical index score of aGVHD mice

**Clinical index score**	**0**	**1**	**2**
Weigh loss	<10%	10%-25%	>25%
Position	Normal	Dorsiflexion at rest	Severe dorsiflexion
Activity	Normal	Slight or moderate reduction	Resting and motionless
Hair texture	Smooth	Slight or moderate bristles	Severely bristly and untidy
Skin integrity	Integrity	Claws or scales of tail fall off	Skin is visibly exposed
