# Supplementary material for: Systematic comparison of hUC-MSCs at various passages reveals the variations of signatures and therapeutic effect on acute graft-versus-host disease
Source: Stem Cell Res Ther. 2019 Nov 28;10:354. doi: 10.1186/s13287-019-1478-4 (PMC6883552; doi:10.1186/s13287-019-1478-4)
Supplement: Supplementary file 5 — Additional file 5: Table S1. The pathological index scores of aGVHD mice including the liver, lung and skin. [file 13287_2019_1478_MOESM5_ESM.docx]

**Table 1 Pathological index scores of aGVHD mice**

|  | **Pathological index score** | **0** | **1** | **2** | **3** |
| --- | --- | --- | --- | --- | --- |
| **Liver** | Inflammation of porta hepatis | Normal | Lymphocyte aggregation around some portals | Lymphocyte aggregation around all the blood vessels | Lymphocyte aggregation around all the blood vessels |
|  | Biliary tract inflammation | Normal | Lymphocyte infiltration in less than 1/3 of the biliary tracts | Lymphocyte infiltration in 1/3-2/3 of the biliary tracts | Lymphocyte infiltration in more than 1/3 of the biliary tracts |
|  | Necrotizing inflammation of the portal vein | Normal | Limited changes of the periportal venous plate in some portal areas | Diffuse changes of periportal venous plate in some portal areas | Diffuse changes of periportal venous plate in all portal areas |
|  | Lobular necrotizing inflammation | Normal | Less than one necrotic lesion per lobule | One necrotic lesion per lobule at least | More than one necrotic lesion per lobule |
|  | Fusion necrosis | Normal | Only in a single lobule | in several lobules | In most of the lobules |
|  | Endothelial inflammation | Normal | Only around portal vein or central lobular vein | Limited around portal vein or central lobular vein | lymphocyte infiltration at least one portal vein or central vein in the lobule |
|  | Lymphocyte infiltration in the hepatic sinus | Normal | Single lobule | In some lobules | In most of the lobules |
| **Lung** | Lymphocyte infiltration in the airway and perivascular lumen | Normal | Lymphocyte cannulated infiltration of 1-3 cells in diameter | Lymphocyte cannulated infiltration of 4-10 cells in diameter | Lymphocyte cannulated infiltration of more than 10 cells in diameter |
|  | Lymphocyte infiltration of alveoli or interstitial tissue | Normal | Lymphocyte infiltration is only seen under high power microscopy | Interstitial tissue thickening | Interstitial tissue thickening, Alveolar lymphocyte infiltration |
|  | Percentage of lung tissue damage | <5% | 5%-25% | 25%-50% | >50% |
| **Skin** | Damage or inflammation | Normal | Balloon-like changes of keratinocytes in basal layer | Inflammatory cell infiltration in sebaceous glands and appendages | Epidermal absence |
