# Supplementary material for: Systematic comparison of hUC-MSCs at various passages reveals the variations of signatures and therapeutic effect on acute graft-versus-host disease
Source: Stem Cell Res Ther. 2019 Nov 28;10:354. doi: 10.1186/s13287-019-1478-4 (PMC6883552; doi:10.1186/s13287-019-1478-4)
Supplement: Supplementary file 7 — Additional file 7. The details accompanied with the main manuscript including Additional Figure Legends and Additional Tables were listed. [file 13287_2019_1478_MOESM7_ESM.docx]

**Systematic Comparison of hUC-MSCs at Various Passages Reveals the Variations of Signatures and Therapeutic Effect on Acute Graft-versus-host Disease**

Qinjun Zhao^1,2^**^§^**, Leisheng Zhang^1-6^**^§*^**, Yimeng Wei^1^, Hao Yu^2^, Linglin Zou^7^, Jiali Huo^1^, Hongju Yang^8^, Baoquan Song^9^, Teng Wei^10^, Dan Wu^1^, Wenxia Zhang^1^, Lei Zhang^1^, Dengke Liu^4^, Zongjin Li^3^, Ying Chi^1^, Zhibo Han^1,2*^, Zhongchao Han^1,2,5*^

**Additional Figure Legends for Addition Fig S1-S4;**

**Additional Tables 1-3;**

**Figure S1. Relative to Figure 1. Identification of hUC-MSCs at various passages by flow cytometry**

(**a**) Flow cytometry (FCM) analysis of MSC markers (CD73, CD105, CD44) of three independent hUC-MSCs at various passages (P3, P6, P15) cultured in 10% FBS/DMEM/F12. (**b**) One of the representative flow cytometry (FCM) analysis of MSC marker of hUC-MSCs at various passages (P3, P6, P15) cultured in 10% FBS/DMEM/F12. (**c**) Flow cytometry (FCM) analysis of apoptosis (7-AAD, Annex V) in hUCs-MSCs cultured in 10% FBS/DMEM/F12. **(d)** Western blotting analysis of pluripotency markers (OCT4, SOX2, NANOG) in hUC-MSCs at various passages (P3, P6, P15) cultured in 10%FBS/DF12 medium. α-Tubulin was used as a loading control.

**Figure S2. Relative to Figure 2. Identification of mutation spectrum and content of hUC-MSCs at various passages**

(**a-b**) Cumulative analysis of genetic mutations in hUC-MSCs at various passages (P3, P6, P15) based on SNP data of 334378 exons. (**c**) Hierarchical clustering analysis of Fraction of SNV mutations among the indicated hUC-MSCs at various passages (P3, P6, P15). (**d**) hUC-MSCs with different origins exhibited different signatures (Signature A, signature B, signature C). (**e**) Unsupervised hierarchical clustering analysis of the different signatures (Signature A, signature B, signature C) with the 30 kinds of characteristics of somatic point mutations.

**Figure S3. Relative to Figure 3. Identification of tissue distribution and hematopoietic-supporting effect of hUC-MSCs.**

(**a**) Signaling pathway analysis of the mutated genes in hUC-MSCs at various passages (P3, P6, P15) by KEGG website. (**b**) Amplification curve of qRT-PCR analysis based on the signal intensity of fluorescence. The genomic DNA of mice liver cells was used as the standard to detect the *Rapsn* gene, and the genomic DNA of MSCs was used as the standard to detect the human *β-GLOBIN* gene. (**c**) Standard curve of qRT-PCR analysis based on the amounts of cycle and copies amplification curve. (**d**) Content of hUC-MSCs in the tissues of mice (lung, kidney, femur, heart). (**e**) The traditional hematopoietic colony forming unit (CFU) assay. Briefly, after 14 days culture in methylcellulose medium, the PBMC-derived colonies such as CFU-G, CFU-M and CFU-GM were calculated.

**Figure S4. Relative to Figure 5.** **Clinical symptoms and physical index scores of GVHD mice**

(**a**) Clinical symptoms and physical index scores of GVHD mice, including weight loss, position, activity, hair texture and skin integrity.

**Additional Tables**

**Table S1. Primers used in this study.**

Real-time PCR primer sequences.

| Gene | Forward Primer | Reverse Primer |
| --- | --- | --- |
| *ACTIN* | CTCTTCCAGCCTTCCTTCCT | AGCACTGTGTGTTGGCGTACAG |
| *POU5F1* | CTTGAATCCCGAATGGAAAGGG | GTGTATATCCCAGGGTGATCCTC |
| *SOX2* | GCCGAGTGGAAACTTTTGTCG | GGCAGCGTGTACTTATCCTTCT |
| *NANOG* | TTTGTGGGCCTGAAGAAAACT | AGGGCTGTCCTGAATAAGCAG |
| *ADIPOQ* | TGGTCCTAAGGGAGACATCG | TGGAATTTACCAGTGGAGCC |
| *PPAR-γ* | GCTGGCCTCCTTGATGAATA | TGTCTTCAATGGGCTTCACA |
| *FABP4* | ACTGGGCCAGGAATTTGACG | CTCGTGGAAGTGACGCCTT |
| *RUNX2* | CTCACTACCACACCTACCTG | TCAATATGGTCGCCAAACAGATTC |
| *BGLAP* | GGCGCTACCTGTATCAATGG | TCAGCCAACTCGTCACAGTC |
| *COL1A1* | GAGGGCCAAGACGAAGACATC | CAGATCACGTCATCGCACAAC |
| *ACAN* | CCCCTGCTATTTCATCGACCC | GACACACGGCTCCACTTGAT |
| *SOX9* | AATGGAGCAGCGAAATCAAC | CAGAGAGATTTAGCACACTGATC |
| *COL2A1* | TGGACGATCAGGCGAAACC | GCTGCGGATGCTCTCAATCT |

**Table S2 The ELISA kits used in this study.**

The ELISA kits for cytokine detection.

| Kit | Cat. NO. | Source |
| --- | --- | --- |
| hIL-6 | D6050 | R&D Systems |
| hIL-8 | D8000C | R&D Systems |
| hVEGF | DVE00 | R&D Systems |
| hG-CSF | DCS50 | R&D Systems |
| hHGF | DHG00B | R&D Systems |
| hTGF-β1 | DB100B | R&D Systems |
| hTNF-α | DTA00D | R&D Systems |
| hPGE-2 | KGE004B | R&D Systems |

**Table S3. Antibodies used in this study.**

Antibodies for flow cytometry.

| Antibody | Cat. NO. | Source |
| --- | --- | --- |
| Anti-CD11b-PE | 557743 | BD Pharmigen |
| Anti-CD19-APC | 560167 | BD Pharmigen |
| Anti-CD3-Cy7 | 560910 | BD Pharmigen |
| Anti-CD4-PE | 550630 | BD Pharmigen |
| Anti-CD14-PE | 562691 | BD Pharmigen |
| Anti-CD25-APC | 561399 | BD Pharmigen |
| Anti-CD44-PE | 550989 | BD Pharmigen |
| Anti-CD73-Percp-cy5.5 | 46-0739-42 | eBioscience |
| Anti-CD90-FITC | 11-0909-42 | eBioscience |
| Anti-CD105-APC | 323208 | BioLegend |
| Anti-CD127-FITC | 560549 | BD Pharmigen |
| Anti-IFN-γ-BV605 | 745111 | BD Pharmigen |
| Anti-HLA-DR-PE | 565073 | BD Pharmigen |
| Anti-CD31-PE | 560975 | BD Pharmigen |
| Anti-CD34-APC | 555824 | BD Pharmigen |
| Anti-CD45-FITC | 560975 | BD Pharmigen |
| Anti-CD66b-PE | 561650 | BD Pharmigen |

Antibodies for western-blotting assay.

| Name | Company | Catalog | Host | Dilution |
| --- | --- | --- | --- | --- |
| OCT3/4 | Santa Cruz | SC-9081 | Rabbit | 1:1000 |
| SOX2 | Millipore | AB5603 | Rabbit | 1:500 |
| NANOG | Cell Signaling | 3580 | Rabbit | 1:300 |
| α-Tubulin | Abcam | Ab11304 | Mouse | 1:10000 |
